# ERα expression in T lymphocytes is dispensable for estrogenic effects in bone

**DOI:** 10.1530/JOE-18-0183

**Published:** 2018-05-30

**Authors:** K L Gustafsson, K H Nilsson, H H Farman, A Andersson, V Lionikaite, P Henning, J Wu, S H Windahl, U Islander, S Movérare-Skrtic, K Sjögren, H Carlsten, J-Å Gustafsson, C Ohlsson, M K Lagerquist

**Affiliations:** 1 Center for Bone and Arthritis ResearchDepartment of Internal Medicine and Clinical Nutrition, Institute of Medicine, Sahlgrenska Academy, University of Gothenburg, Gothenburg, Sweden; 2 Center for Bone and Arthritis ResearchDepartment of Rheumatology and Inflammation Research, Institute of Medicine, Sahlgrenska Academy, University of Gothenburg, Gothenburg, Sweden; 3 Center for Nuclear Receptors and Cell SignalingDepartment of Biology and Biochemistry, University of Houston, Houston, Texas, USA

**Keywords:** T lymphocytes, estrogen receptor alpha, estrogen, bone loss

## Abstract

Estrogen treatment has positive effects on the skeleton, and we have shown that estrogen receptor alpha (ERα) expression in cells of hematopoietic origin contributes to a normal estrogen treatment response in bone tissue. T lymphocytes are implicated in the estrogenic regulation of bone mass, but it is not known whether T lymphocytes are direct estrogen target cells. Therefore, the aim of this study was to determine the importance of ERα expression in T lymphocytes for the estrogenic regulation of the skeleton using female mice lacking ERα expression specifically in T lymphocytes (Lck-ERα^−/−^) and ERα^flox/flox^ littermate (control) mice. Deletion of ERα expression in T lymphocytes did not affect bone mineral density (BMD) in sham-operated Lck-ERα^−/−^ compared to control mice, and ovariectomy (ovx) resulted in a similar decrease in BMD in control and Lck-ERα^−/−^ mice compared to sham-operated mice. Furthermore, estrogen treatment of ovx Lck-ERα^−/−^ led to an increased BMD that was indistinguishable from the increase seen after estrogen treatment of ovx control mice. Detailed analysis of both the appendicular (femur) and axial (vertebrae) skeleton showed that both trabecular and cortical bone parameters responded to a similar extent regardless of the presence of ERα in T lymphocytes. In conclusion, ERα expression in T lymphocytes is dispensable for normal estrogenic regulation of bone mass in female mice.

## Introduction

Estrogen, the main female reproductive hormone, is a major regulator of bone homeostasis and it is well known that estrogen deficiency after menopause increases fracture risk and that estrogen treatment decreases this risk (Ettinger 1988). However, estrogen is not suitable as treatment due to severe side effects such as increased risk of cancer in reproductive organs and venous thrombosis (Rossouw* et al*. 2002, Barrett-Connor* et al*. 2005, Khosla 2010, Marjoribanks* et al*. 2017). It is therefore important to identify the mechanisms behind the skeletal effects of estrogen to be able to separate the positive bone protective effects from the negative side effects in order to facilitate development of new tissue specific treatments. The effects of estrogen are mediated primarily via the estrogen receptors (ERs), ER alpha (ERα) and ER beta (ERβ), and ERα is considered the main regulator of the protective effects of estrogen in the skeleton (Lindberg* et al*. 2002, Walker & Korach 2004). ERα is expressed in several cell types of mesenchymal origin, including bone forming osteoblasts and osteocytes, and an important role of ERα expression in these cell types for the skeleton has recently been demonstrated (Windahl* et al*. 1999, Almeida* et al*. 2013, Maatta* et al*. 2013, Melville* et al*. 2014). Furthermore, ERα expression in osteoclasts, originating from hematopoietic stem cells, has also been shown to affect the skeleton (Nakamura* et al*. 2007, Martin-Millan* et al*. 2010). Our group has demonstrated that ERα expression in hematopoietic cells is important for a normal estrogen treatment response in bone (Henning* et al*. 2014) and other cells of hematopoietic origin, aside from osteoclasts, have been shown to be involved in the estrogenic regulation of bone mass, including T lymphocytes (Kong* et al*. 1999). Pacifici *et al.* have shown that mice lacking T lymphocytes are protected from bone loss caused by ovariectomy (ovx) (i.e. estrogen deficiency), and they have also shown that ovx enhances T lymphocyte production of tumor necrosis factor alpha (TNFα), leading to increased bone loss (Kong* et al*. 1999, Cenci* et al*. 2000). Thus, T cells are strongly implicated in the estrogenic regulation of the skeleton, but it is not known whether T lymphocytes are direct estrogen target cells or if the involvement of T lymphocytes is indirect via estrogen signaling in other cells. The aim of this study was therefore to determine if T lymphocytes are direct estrogen target cells by evaluating the importance of ERα expression specifically in T lymphocytes for the estrogenic regulation of bone mass in female mice.

## Materials and methods

### Animals

All experimental procedures involving animals were approved by the Ethics Committee at the University of Gothenburg. The mice were housed in a standard animal facility under controlled temperature (22°C) and photoperiod (12 h of light and 12 h of darkness) and fed phytoestrogen-free pellet diet (R70, Lactamin AB, Sweden) and tap water *ad libitum*. To generate T lymphocyte-specific ERα-inactivated mice, ERα^flox/flox^ mice on C57BL/6 background (Antonson* et al*. 2012), in which exon 3 of the ERα (*Esr1*) gene is flanked by loxP sequences, were crossed with Lck-Cre mice (Hennet* et al*. 1995) on C57BL/6 background to generate Lck-Cre;ERα^flox/+^ mice. The proximal Lck promoter has previously been used to promote T lymphocyte-specific gene inactivation (Pacifici 2012, Weitzmann 2017) and the promoter is turned on in the earliest thymic immigrants and in all subsequent T cell lineages (Onal* et al*. 2012). The Lck-Cre;ERα^flox/+^ mice were crossed with ERα^flox/flox^ mice to generate conditional mutants (Lck-Cre;ERα^flox/flox^, hereafter referred to as Lck-ERα^−/−^) and corresponding littermate controls (ERα^flox/flox^). The total body areal bone mineral density (aBMD) was similar between Cre-positive and Cre-negative ERα^flox/−^ mice (50.3 ± 0.5 and 49.3 ± 0.7; mg/cm^2^), demonstrating that the Cre-construct itself does not affect bone mass. Genotyping of the mice was performed by PCR using Cre primers P1: 5′-GTT CGC AAG AAC CTG ATG GAC A-3′ and P2: 5′-CTA GAG CCT GTT TTG CAC GTT C-3′ and ERα^flox/flox^ primers P3: 5′-GGA ATG AGA CTT GTC TAT CTT CGT-3′ and P4: 5′-GAC ACA TGC AGC AGA AGG TA-3′. Gonadal intact female mice, 2–7 months of age, were used for measuring ERα mRNA expression in different tissues and for extraction of CD3-positive cells from thymus. Twelve-week-old female Lck-ERα^−/−^ mice and control littermates were ovariectomized (ovx) and treated with a subcutaneous slow-release pellet (60-day-release pellet, Innovative Research of America) with 17β-estradiol (E2, 167 ng/mouse/day) or placebo for 4 weeks or sham-operated and treated with placebo for 4 weeks. Surgery was performed under anesthesia with isoflurane (Baxter Medical AB, Kista, Sweden) and Rimadyl (Orion Pharma AB, Animal Health, Sollentuna, Sweden) was given postoperatively as an analgesic. At termination, the mice were anesthetized with Ketanest/Dexdomitor (Pfizer/Orion Pharma), bled and killed by cervical dislocation. Hypothalamus, uterus, fat depots and muscle were collected, weighed and snap-frozen. The femur and vertebrae L5 were dissected, fixed in 4% paraformaldehyde and stored for further analysis.

### CD3-positive cell separation in thymus

CD3-positive cells were extracted from freshly dissected thymus with a mouse CD3ε MicroBead Kit (MACS, Miltenyi Biotec) according to the manufacturer’s protocol.

### Real-time PCR

RNA was isolated from hypothalamus, fat, muscle and bone marrow from long bones (tibia and femur) using the RNeasy Mini Kit (Qiagen). RNA from cortical bone was isolated using TRIzol reagent (Sigma) followed by the RNeasy Mini Kit (Qiagen). Amplifications were performed using the Applied Biosystem StepOnePlus Real-Time PCR System (PE, Applied Biosystems) and Assay-on-Demand primer and probe sets (PE, Applied Biosystems), labeled with the reporter fluorescent dye FAM. Predesigned primers and probe labeled with the reporter fluorescent dye VIC, specific for 18S ribosomal RNA, were included in the reaction as an internal standard. The assay identification number for *Esr1* was Mm00433147_m1.

### Assessment of bone parameters

#### Dual-energy X-ray absorptiometry

Analysis of total body areal bone mineral density (aBMD) and lumbar spine aBMD (vertebrae L_2_–L_5_) was performed using a Lunar PIXImus mouse densitometer (Wipro GE Healthcare).

#### High-resolution microcomputed tomography

High-resolution microcomputed tomography (μCT) analysis was performed on the vertebrae L_5_ and femur using an 1172 model μCT (Bruker MicroCT, Aartselaar, Belgium) as previously described (Moverare-Skrtic* et al*. 2014). The vertebrae and femur were imaged with an X-ray tube voltage of 49 kV, a current of 200 μA and with a 0.5 mm aluminum filter. The scanning angular rotation was 180°, and the angular increment was 0.70°. The voxel size was 4.49 mm isotropically. NRecon (version 1.6.9) was used to perform the reconstruction after the scans. In the vertebrae, the trabecular bone in the vertebral body caudal of the pedicles was selected for analysis within a conforming volume of interest (cortical bone excluded) commencing at a distance of 4.5 μm caudal of the lower end of the pedicles, and extending a further longitudinal distance of 225 μm in the caudal direction. In the femur, the trabecular bone proximal to the distal growth plate was selected for analyses within a conforming volume of interest (cortical bone excluded), commencing at a distance of 650 μm from the growth plate and extending a further longitudinal distance of 134 μm in the proximal direction. The cortical measurements in femur were performed in the mid-diaphyseal region of femur starting at a distance of 5.2 mm from the growth plate and extending a further longitudinal distance of 134 μm in the proximal direction.

### Serum biomarkers

As a marker of bone resorption, serum levels of C-terminal type I collagen fragments were assessed using an ELISA RatLaps kit (CTX-I, Immunodiagostic Systems, Copenhagen, Denmark). Serum levels of procollagen type I N propeptide (P1NP, Immunodiagostic Systems) were analyzed as a marker of bone formation.

### Statistical analyses

Values are given as mean ± s.e.m. Statistical significance was determined using Student’s *t* test. To determine the occurrence of significant differences in the E2 response between Lck-ERα^−/−^ and controls, the interaction *P* value from a two-way ANOVA was used.

## Results

### Generation of mice lacking ERα expression in T lymphocytes

**Figure 1:**
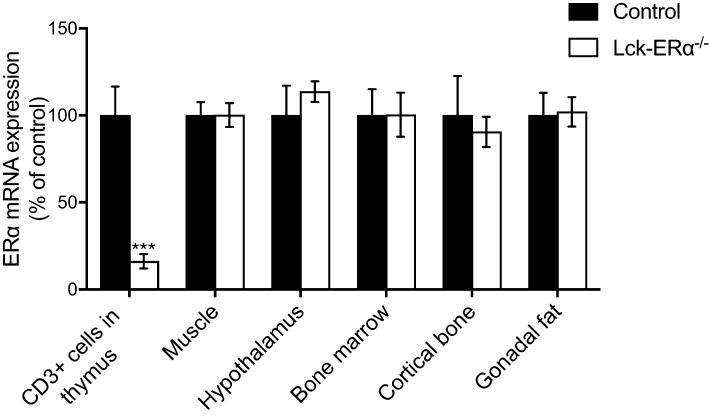
Specific inactivation of ERα mRNA expression in T lymphocytes. Two- to seven-month-old gonadal intact female mice were used to study ERα mRNA expression in CD3-positive cells (T lymphocytes) from thymus, muscle, hypothalamus, bone marrow, cortical bone and gonadal fat. Values are given as mean ± s.e.m. (*n* = 6–13). ***P < 0.001, Student’s *t* test, Lck-ERα^−/−^ vs controls.

To generate mice lacking ERα expression in T lymphocytes, we used the Cre-loxP system. The effectiveness of ERα gene inactivation was demonstrated by an 84% reduction in ERα mRNA levels in thymic T lymphocytes (CD3-positive cells) in Lck-ERα^−/−^ mice compared to controls (Fig. 1). ERα mRNA expression was also measured in muscle, hypothalamus, bone marrow, cortical bone and gonadal fat, and no differences were seen between Lck-ERα^−/−^ and controls in these tissues (Fig. 1), confirming a specific impairment of ERα expression in T lymphocytes.

### Deletion of ERα expression in T lymphocytes does not affect bone mass or alter bone loss caused by estrogen deficiency

Uterine weights did not differ between sham-operated Lck-ERα^−/−^ and control mice (Table 1). Thymus weights were also unaffected by ERα inactivation in T lymphocytes (Table 1). Total body aBMD and lumbar spine aBMD, analyzed by dual-energy X-ray absorptiometry (DXA), were similar between Lck-ERα^−/−^ and control mice (Table 1). Furthermore, analysis of trabecular bone (BV/TV; bone volume/total volume, Tb.N.; trabecular number, Tb.Th.; trabecular thickness and Tb.Sp.; trabecular separation) in vertebrae (L_5_) revealed no significant differences between Lck-ERα^−/−^ and control mice (Table 1). Trabecular (BV/TV, Tb.N., Tb.Th. and Tb.Sp.) and cortical (Ct.Th.; cortical thickness, Endo. C.; endosteal circumference and Peri. C.; periosteal circumference) bone parameters in femur were also similar between Lck-ERα^−/−^ and control mice (Table 1 and Supplementary Fig. 1, see section on supplementary data given at the end of this article). In addition, serum levels of biomarkers for bone formation (P1NP) and bone resorption (CTX-I) were similar between Lck-ERα^−/−^ and control mice (Table 1).

**Table 1:** Body and skeletal characteristics of 16-week-old sham-operated Lck-ERα^−/−^ and control mice.

	Control	Lck-ERα^−/−^
Body weight (g)	21.8 ± 0.5	22.8 ± 0.7
Uterus weight/bw (mg/g)	2.9 ± 0.3	3.2 ± 0.3
Thymus weight/bw (mg/g)	2.5 ± 0.1	2.6 ± 0.1
Total body aBMD (mg/cm^2^)	49.8 ± 0.5	49.2 ± 0.4
Lumbar spine aBMD (mg/cm^2^)	56.8 ± 1.4	56.3 ±1.0
Vertebra, L_5_		
Bone volume/total volume (BV/TV; %)	22.9 ± 0.7	24.5 ± 0.6
Trabecular number (Tb.N; 1/mm)	4.9 ± 0.1	5.3 ± 0.1
Trabecular thickness (Tb.Th; μm)	46.4 ± 0.8	46.5 ± 0.6
Trabecular separation (Tb.Sp; μm)	152 ± 2.5	147 ± 3.4
Femur		
Bone volume/total volume (BV/TV; %)	14.7 ± 0.8	16.1 ± 0.8
Trabecular number (Tb.N; 1/mm)	3.3 ± 0.2	3.6 ± 0.2
Trabecular thickness (Tb.Th; μm)	44.6 ± 1.0	45.0 ± 1.3
Trabecular separation (Tb.Sp; μm)	118 ± 2.0	116 ± 2.0
Cortical thickness (Ct.Th; μm)	186 ± 2.5	188 ± 3.0
Endosteal circumference (Endo. C; mm)	3.40 ± 0.04	3.32 ± 0.04
Periosteal circumference (Peri. C; mm)	4.57 ± 0.04	4.50 ± 0.05
Serum biomarkers		
P1NP (ng/mL)	73.6 ± 4.4	85.7 ± 5.7
CTX-I (ng/mL)	16.2 ± 1.9	17.8 ± 2.3

Values are given as mean ± s.e.m. (*n* = 9–10). Student’s *t* test, Lck-ERα^−/−^ vs controls.

CTX-I, C-terminal type I collagen fragments; P1NP, procollagen type I N propeptide.

Uterine weights were significantly decreased to a similar extent after ovariectomy (ovx) in control (−83 ± 0.9%, *P* < 0.001) and Lck-ERα^−/−^ (−85 ± 0.7%, *P* < 0.001) mice confirming successful ovx. Ovx resulted in a similar increase in thymus weight in control (42 ± 3.6%, *P* < 0.001) and Lck-ERα^−/−^ (32 ± 4.5%, *P* < 0.001) mice compared to sham-operated mice. Furthermore, total body aBMD was decreased after ovx to a similar extent in control (−4.2 ± 1.4%, *P* < 0.05) and Lck-ERα^−/−^ mice (−5.7 ± 0.7% *P* < 0.001) as compared to sham-operated mice.

### ERα expression in T lymphocytes is not required for a normal estrogenic response in bone

**Figure 2:**
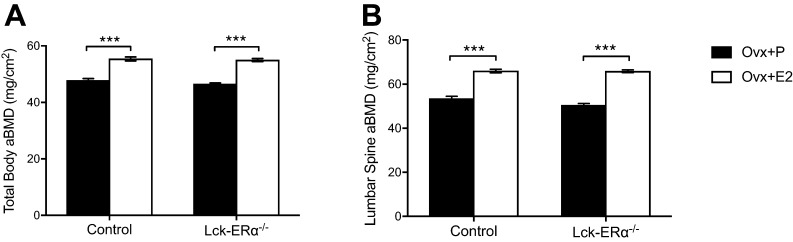
The estrogen response in total body areal bone mineral density is not mediated via estrogen receptor alpha (ERα) in T lymphocytes. Twelve-week-old Lck-ERα^−/−^ and control mice were ovariectomized and treated with 17β-estradiol (E2, 167 ng/mouse/day) or placebo (P) for 4 weeks. Total body areal bone mineral density (aBMD) (A) and lumbar spine aBMD (B) were measured by DXA. Values are given as mean ± s.e.m. (*n* = 9–10). ***P < 0.001, Student’s *t* test, E2 vs placebo treatment.

**Figure 3:**
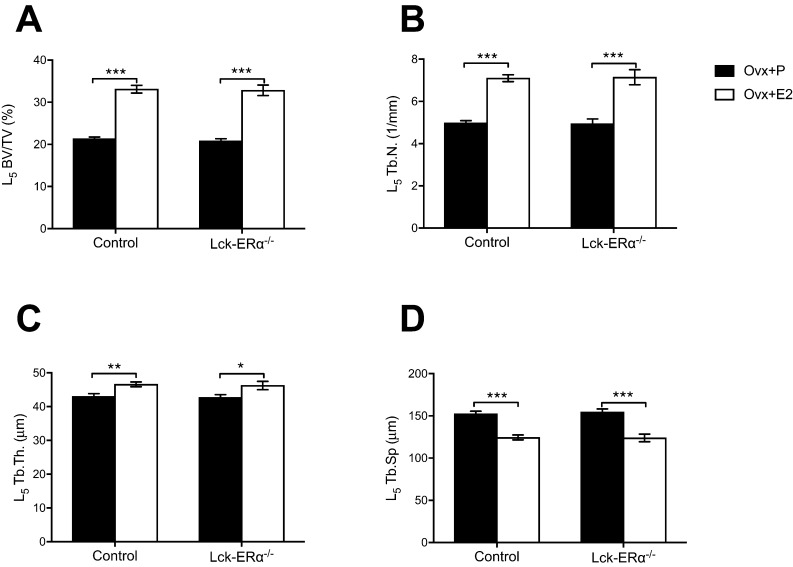
The estrogen response in trabecular bone is not mediated via estrogen receptor alpha (ERα) in T lymphocytes. Twelve-week-old Lck-ERα^−/−^ and control mice were ovariectomized and treated with 17β-estradiol (E2, 167 ng/mouse/day) or placebo (P) for 4 weeks. Bone volume per tissue volume (BV/TV) (A), trabecular number (Tb.N.) (B), trabecular thickness (Tb.Th.) (C) and trabecular separation (Tb.Sp.) (D) were analyzed in vertebrae L_5_ using high-resolution microcomputed tomography (μCT). Values are given as mean ± s.e.m. (*n* = 9–10). *P < 0.05, **P < 0.01, ***P < 0.001, Student’s *t* test, E2 vs placebo treatment.

**Figure 4:**
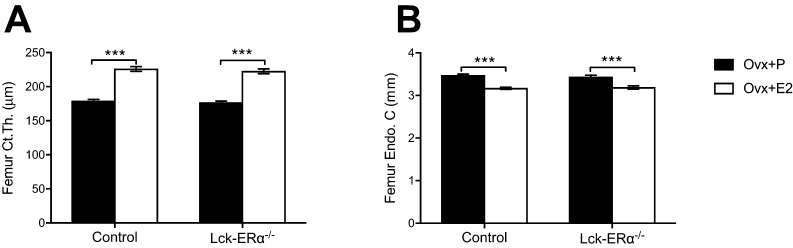
The estrogen response in cortical bone is not mediated via estrogen receptor alpha (ERα) in T lymphocytes. Twelve-week-old Lck-ERα^−/−^ and control mice were ovariectomized and treated with 17β-estradiol (E2, 167 ng/mouse/day) or placebo (P) for 4 weeks. Cortical thickness (Ct.Th.) (A) and endosteal circumference (Endo C.) (B) were analyzed in femur using high-resolution microcomputed tomography (μCT). Values are given as mean ± s.e.m. (*n* = 9–10). ***P < 0.001, Student’s *t* test, E2 vs placebo treatment.

E2 treatment increased uterine weights and decreased thymus weights to a similar extent in Lck-ERα^−/−^ and control mice compared to vehicle-treated mice (Table 2). Skeletal analyses demonstrated a similar increase in total body aBMD and lumbar spine aBMD after estrogen treatment in Lck-ERα^−/−^ and control mice compared to vehicle treatment (Fig. 2A and B). Estrogen treatment increased trabecular BV/TV in vertebrae L_5_ to a similar extent in Lck-ERα^−/−^ mice and controls (Fig. 3A). Furthermore, analysis of microstructural parameters in vertebral trabecular bone revealed no significant differences in the response to estrogen treatment between Lck-ERα^−/−^ mice and controls in regards to trabecular number (Tb.N.), trabecular thickness (Tb.Th.) and trabecular separation (Tb.Sp.) (Fig. 3B, C and D). The estrogen treatment response on trabecular bone parameters (BV/TV, Tb.N., Tb.Th. and Tb.Sp.) in the appendicular skeleton, analyzed in femur, was also similar in Lck-ERα^−/−^ mice and controls (Table 2 and Supplementary Fig. 2). Cortical bone parameters were determined in the mid-diaphyseal region of femur and estrogen resulted in a similar increase in cortical thickness (Ct.Th.) and a similar decrease in endosteal circumference (Endo. C.) in Lck-ERα^−/−^ mice and controls (Fig. 4A, B and Supplementary Fig. 2).

**Table 2:** The estrogen response on organ weights and trabecular bone parameters in femur are not mediated via estrogen receptor alpha (ERα) in T lymphocytes.

	Control		Lck-ERα^−/−^	
	Ovx + P	Ovx + E2	Ovx + P	Ovx + E2
Uterus weight/bw (mg/g)	0.50 ± 0.03	8.52 ± 0.95***	0.46 ± 0.03	7.12 ± 0.75***
Thymus weight/bw (mg/g)	3.45 ± 0.10	0.41 ± 0.04***	3.33 ± 0.14	0.54 ± 0.13***
Femur				
Bone volume/total volume (BV/TV; %)	13.0 ± 0.5	55.9 ± 1.9***	12.4 ± 0.6	48.8 ± 3.3***
Trabecular number (Tb.N; 1/mm)	3.2 ± 0.1	13.0 ± 0.3***	3.0 ± 0.1	11.4 ± 1.0***
Trabecular thickness (Tb.Th; μm)	40.8 ± 0.6	43.0 ± 1.6	41.3 ± 0.8	43.8 ± 1.2
Trabecular separation (Tb.Sp; μm)	120 ± 1.3	45.1 ± 2.0***	123 ± 0.9	54.4 ± 5.6***

12-week-old Lck-ERα^−/−^ and control mice were ovariectomized (ovx) and treated with 17β-estradiol (E2, 167 ng/mouse/day) or placebo (P) for 4 weeks. Values are given as mean ± s.e.m. (*n* = 9–10).

***P < 0.001, Student’s *t* test, E2 vs placebo treatment.

## Discussion

It is well established that estrogen has positive effects on bone, but also that it is not suitable as an anti-resorptive osteoporosis treatment due to adverse side effects (Rossouw* et al*. 2002, Barrett-Connor* et al*. 2005, Khosla 2010, Marjoribanks* et al*. 2017). To be able to develop new estrogen-like drugs against bone loss, which lack negative estrogenic effects, it is important to increase the knowledge regarding mechanisms behind the effects of estrogen on bone. We have recently showed that ERα expression in hematopoietic cells is required for a normal estrogenic treatment response in bone (Henning* et al*. 2014), but it was not established, which cell type of hematopoietic origin that was involved in these effects. It is demonstrated that ERα expression in osteoclasts, derived from the hematopoietic lineage, is involved in the regulation of trabecular bone (Nakamura* et al*. 2007, Martin-Millan* et al*. 2010). However, several other cell types of hematopoietic origin are also implicated to be involved in estrogenic regulation of bone mass, including T lymphocytes (Weitzmann & Pacifici 2007).

Estrogen deficiency leads to increased thymic outflow of naïve T lymphocytes and increased activation of T cells (Pacifici 2012, Weitzmann 2017). These events lead to enhanced production of TNFα, which results in increased differentiation and activation of osteoclasts (Cenci* et al*. 2000) and are suggested to be part of the mechanism behind bone loss caused by estrogen deficiency. Thus, presence of estrogen suppresses T lymphocyte-associated deleterious effects on the skeleton. However, it is not established whether these effects are direct, via effects on estrogen signaling in T lymphocytes, or indirect, via estrogenic effects on other cell types, and this is addressed in the present study. If one hypothesizes that the role of T lymphocytes in estrogen deficiency-induced bone loss involves direct alteration of ERα signaling in T lymphocytes, inactivation of ERα expression in T lymphocytes would lead to a decreased estrogenic suppression of T lymphocyte-associated skeletal effects and thereby decreased bone mass.

To investigate this, we have generated mice with impaired ERα expression specifically in T lymphocytes and studied the effects on bone mass and also how the skeleton responds to estrogen deficiency and estrogen treatment.

Analysis of the skeleton in mice lacking ERα expression in T lymphocytes showed no significant difference in total body aBMD compared to littermate controls. Trabecular bone parameters in both femur and vertebrae were also unchanged by T lymphocyte-specific inactivation of ERα, as was cortical bone parameters measurements in the mid-diaphyseal part of femur. In addition, serum levels of biomarkers for bone formation and bone resorption were unaffected by T lymphocyte-specific inactivation of ERα. These data suggest that the ERα in T lymphocytes is dispensable for normal regulation of bone mass in female mice.

As expected, estrogen deficiency, induced by ovariectomy, led to decreased total body aBMD in control mice. Ovariectomy led to a similar decrease in total body aBMD in mice lacking ERα expression in T lymphocytes, suggesting that ERα signaling in T lymphocytes is dispensable for bone loss caused by estrogen deficiency. These results support the previous study by Lee* et al*. showing that T lymphocyte-deficient mice have a normal ovariectomy-induced bone loss (Lee* et al*. 2006). B lymphocytes have also been implicated in bone loss caused by estrogen deficiency, and deletion of RANKL expression in B lymphocytes blunted bone loss in ovariectomized mice (Onal* et al*. 2012). However, it was recently shown that this was not dependent on ERα expression in B lymphocytes since mice with a specific deletion of ERα in B lymphocytes experienced a similar bone loss as normal controls (Fujiwara* et al*. 2016). Thus, ERα signaling in both T and B lymphocytes is dispensable for bone loss caused by estrogen deficiency.

As previously shown by us and others (Lindberg* et al*. 2002, Sims* et al*. 2003, Borjesson* et al*. 2011), E2 treatment increased total body aBMD and both trabecular and cortical bone parameters in ovariectomized control mice. Interestingly, and in contrast to the requirement of ERα expression in T lymphocytes for the ameliorating effects of pharmacological E2 treatment on experimental autoimmune encephalomyelitis (Lelu* et al*. 2011), the skeletal response to estrogen treatment in mice lacking ERα expression in T lymphocytes did not differ from the response in control mice. Thus, the beneficial effects of E2 treatment on bone does not require direct interaction of E2 with ERα in T lymphocytes.

Osteoclasts, which are of hematopoietic origin, have been shown to respond to estrogen directly via ERα, and it is demonstrated that ERα expression in osteoclasts is important for the regulation of trabecular bone in females (Nakamura* et al*. 2007, Martin-Millan* et al*. 2010). Regarding cortical bone, the available evidence suggests that the protective effects of estrogen is mediated mainly via direct actions on mesenchymal cells (Almeida* et al*. 2013, 2017, Manolagas* et al*. 2013, Ucer* et al*. 2016). However, our previous finding (Henning* et al*. 2014) suggests that part of the estrogenic protection of cortical bone is mediated via direct estrogen action in hematopoietic cells. Since inactivation of ERα in osteoclasts has been suggested not to affect cortical bone in females, further studies are needed to determine which other hematopoietic cell type is involved.

Estrogen treatment is known to induce thymic atrophy and global inactivation of ERα results in impaired thymic atrophy after estrogen treatment (Lindberg* et al*. 2002), demonstrating that ERα, at least partly, is involved in mediating this atrophic effect. The effect of estrogen treatment on thymus weight in our study was unaffected by inactivation of ERα in T lymphocytes, demonstrating that the ERα-mediated effect on thymic atrophy is independent of ERα signaling in T lymphocytes. Thus, the atrophic effect of estrogen is most probably mediated via other cells in the thymus and epithelial cells in the thymus stroma have previously been implicated (Staples* et al*. 1999).

Taken together, these data demonstrate that ERα expression in T lymphocytes is not required for a normal response to estrogen treatment neither in trabecular nor in cortical bone in ovariectomized female mice. Thus, our data suggest that T lymphocytes are not a direct target cell for the protective effects of estrogen on bone and implicate other cell types as primary estrogen-responsive cells mediating the positive estrogenic effects on bone. In conclusion, our data suggest that ERα expression in T lymphocytes is dispensable for ovariectomy-induced bone loss and response to estrogen treatment in bone after ovariectomy.

## Supplementary Material

Supporting Figure 1

Supporting Figure 2

## Declaration of interest

The authors declare that there is no conflict of interest that could be perceived as prejudicing the impartiality of the research reported.

## Funding

The Swedish Research Council (grant number 2017-01286); the Swedish Foundation for Strategic Research; the ALF/LUA research grant from the Sahlgrenska University Hospital (grant number ALFGBG-721581); the Gustaf V 80-year fund (grant number FAI-2016-0286); the Swedish Rheumatism Association (grant number R-754891); the Lundberg Foundation (grant number 2017-0076); the Torsten and Ragnar Söderberg’s Foundations (grant number M133/12); the Knut and Alice Wallenberg Foundation (grant number KAW 2015.0317) and the Novo Nordisk Foundation (grant number NNF17Obib26844). J A G is thankful to the Robert A. Welch Foundation for a grant (E-0004).

## Author contribution statement

K L G, C O and M K L conducted the study design. K L G, M K L, K N, A A, H F, V L, P H, J W, U I, S H W, S M S and K S were responsible for acquisition of data and K L G, M K L, J Å G and C O performed the analysis and interpretation of data. M K L, K L G and C O wrote the main manuscript text and K L G and M K L prepared the figures. All authors reviewed the manuscript.
