# Supplementary figures and images for: ERα expression in T lymphocytes is dispensable for estrogenic effects in bone
Source: J Endocrinol. 2018 May 30;238(2):129–36. doi: 10.1530/JOE-18-0183 (PMC6026922; doi:10.1530/JOE-18-0183)

Control

Lck-ER $\alpha$ <sup>-/-</sup>

A

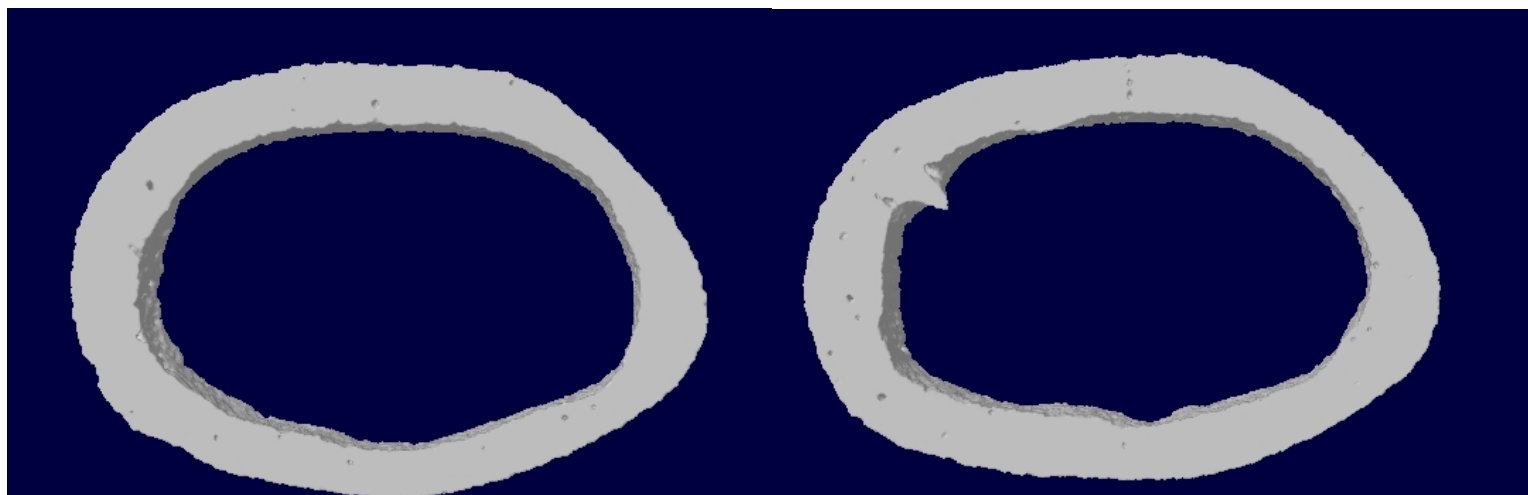

B

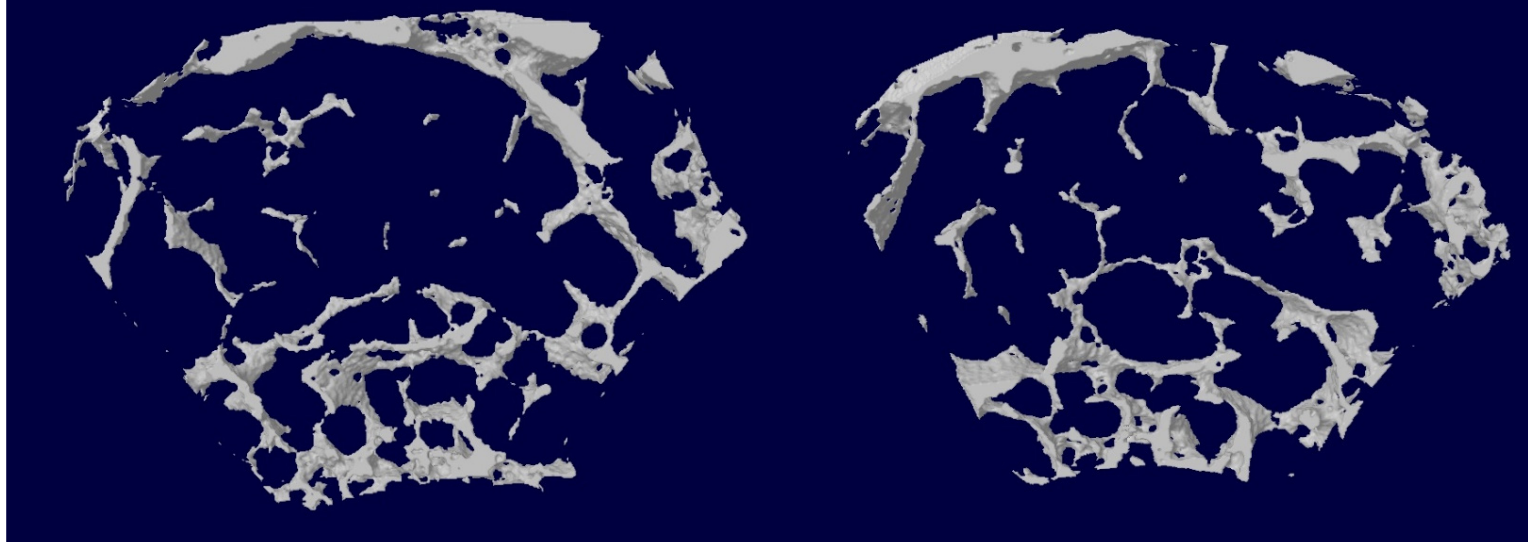

Supplement: Supporting Figure 1 [file joe-238-129-s001.pdf]

Control

Lck-ER $\alpha^{-/-}$

P

E2

P

E2

A

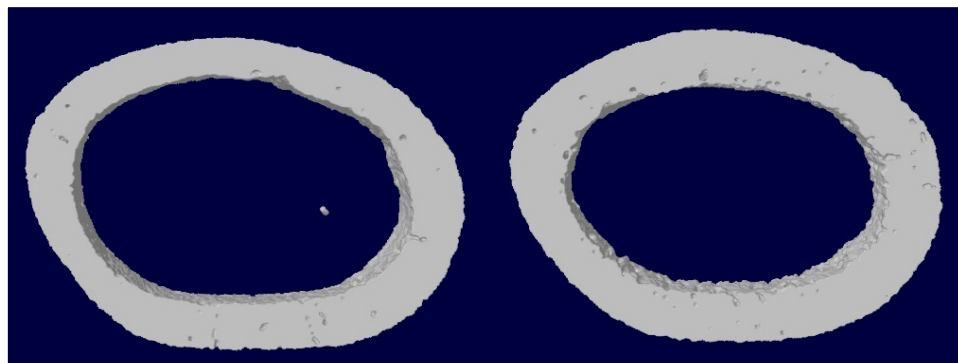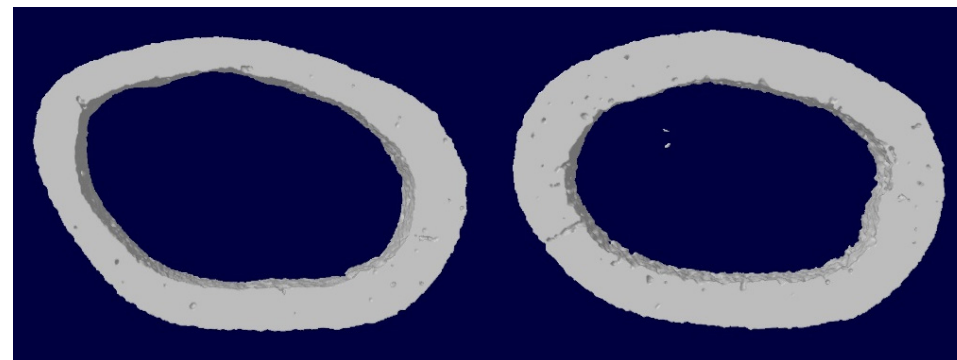

B

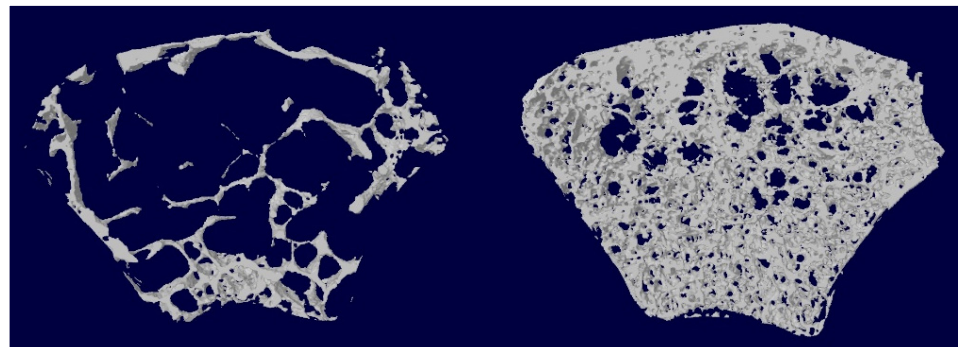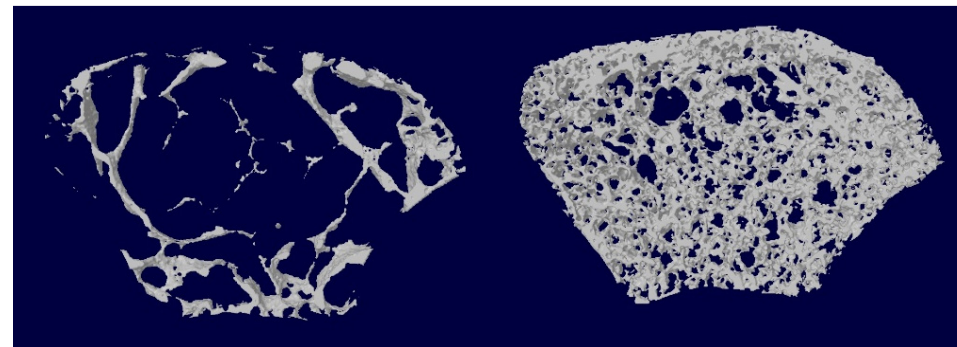

Supplement: Supporting Figure 2 [file joe-238-129-s002.pdf]
